# Development and Evaluation of Copper Electrodes, Modified with Bimetallic Nanoparticles, to be Used as Sensors of Cysteine-Rich Peptides Synthesized by Tobacco Cells Exposed to Cytotoxic Levels of Cadmium

**DOI:** 10.3390/molecules24122200

**Published:** 2019-06-12

**Authors:** Carlos Velasco-Medina, Patricio J. Espinoza-Montero, Marjorie Montero-Jimenez, José Alvarado, Mónica Jadán, Patricio Carrera, Lenys Fernandez

**Affiliations:** 1 Escuela de Ciencias Químicas, Pontificia Universidad Católica del Ecuador, Avenida 12 de Octubre y Roca, P.O.Box 17-01-2184, Quito, Ecuador; carlos.velasco@epn.edu.ec (C.V.-M.); marjorie_cpp@hotmail.com (M.M.-J.); 2 Facultad de Ingeniería Química y Agroindustria, Escuela Politécnica Nacional, Ladrón de Guevara E11-253, Quito, Ecuador; 3 Departamento de Química, Universidad Simón Bolívar, Apartado 89000, Caracas, Venezuela; jalvar@usb.ve; 4 Departamento de Ciencias de la Vida y de la Agricultura, Universidad de las Fuerzas Armadas ESPE, Laboratorio de Cultivo de Tejidos Vegetales, Grupo BIOCEMP, Av. General Rumiñahui s/n, Sangolqui, P.O.Box 171-5-231B, Ecuador; mbjadan@espe.edu.ec; 5 Hidroecuador, Apartado EC1701, Quito, Ecuador; info@nanoinstrumentos.net

**Keywords:** Brdička reaction, electrochemical detection, cysteine-rich peptides, bimetallic deposits, heavy metal pollution

## Abstract

We report on two new electrochemical sensors which, coupled to differential pulse voltammetry, constitutes a useful tool for diagnosis of heavy metal pollution. The electrochemical sensors AgHgNf/Cu and the AgBiNf/Cu were obtained by deposition of bimetallic particles of AgHg or AgBi on copper electrodes covered with a Nafion (Nf) film, respectively. Micrographs of the electrode’s surface showed evenly scattered bimetallic particles, with an approximate diameter of 150 nm, embedded in the Nafion (Nf) film. In order to test the electrodes, the hydrogen evolution signal according to the Brdička reaction was measured for the determination of cysteine-rich peptides (CRp) produced by plants. To check the accuracy of the electrodes, real samples of *Nicotiana tabacum* cells exposed to cytotoxic levels of cadmium were tested. The AgHgNf/Cu electrode produced detection limits (DLs) of 0.088 µmol L^−1^ for Cysteine and 0.139µmol L^−1^ for Glutathione, while for the AgBiNf/Cu electrode DLs were 0.41 µmol L^−1^ for cysteine and 0.244 µmol L^−1^ for glutathione. Thus, the new electrodes could be a useful analytical electrochemical system very convenient for fieldwork. The electrodes were capable of direct, accurate, and sensitive detection of synthesized peptides, despite the complex matrix where the *Nicotiana tabacum* cells were grown.

## 1. Introduction

**Figure 1:**
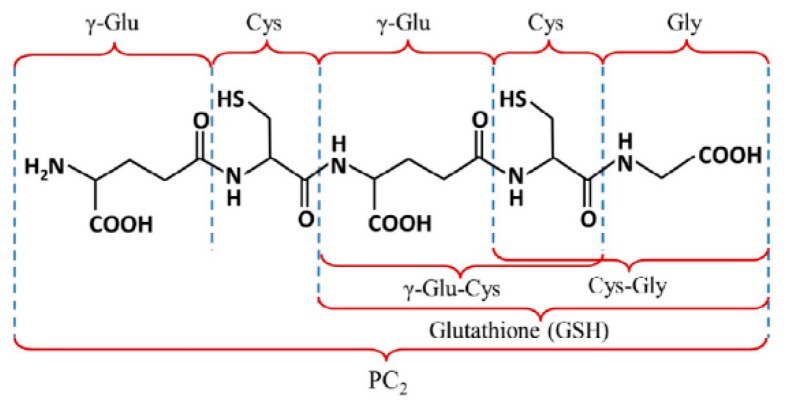
Chemical structure of phytochelatin CRp2 [4].

Currently, heavy metal pollution is a serious environmental problem that is affecting animals and plants. Fortunately, plants have developed protection mechanisms against heavy metals’ toxic effects. One of those mechanisms is the synthesis of cysteine-rich peptides (CRp) such as glutathione, glutathione oxide, metallothionein, and phytochelatin. Glutathione (GSH) and those other peptides contain the γ-glutamate-cysteine-glycine tripeptide, usually known as the Glu-Cys-Gly unit, which makes them effective in encapsulating heavy metals by means of the (-SH) cysteine group action. Since metal´s toxicity affects the cytoplasm during its passage across to the vacuole where they are stored, encapsulation prevents direct contact of the metals with the tissue, thus minimizing damage [1]. Cysteine-rich peptides extensions such as phytochelatin (Figure 1), contain between 2 and 11 repetitive units of γ-Glu-Cys ending on a Gly terminal or contain 2 or 3 unit chains of glutathione, which could act in the encapsulation process. Generation of CRp has been studied in a wide variety of plants and microorganisms. It has been shown that CRp could be taken as a response to heavy metal contamination and it is understood that the best trigger of cysteine-rich peptides generation is Cd, followed by Pb, Hg, and Zn [2,3].

Study of CRp and its phytoremediation capability could be carried out using techniques like chromatography, capillary electrophoresis, or electrochemical methods. Usually, the application of those techniques emphasizes the need for separation and extraction of the chelates from a complex matrix [4,5,6]. The electrochemical behavior of CRp could be studied and the peptides could be determined without interference from vegetal matrices applying the Brdička reaction using conventional Hg electrodes. The Brdička reaction is based on the interaction of cobalt salts and cysteine thiol groups, forming a complex that activates the cysteine hydrogen atom and catalyzes hydrogen evolution at negative potentials [7,8,9]. Determination using mercury electrodes has the drawbacks that Hg is environmentally unfriendly due to the fact that the Hg electrode has poor mechanical stability and Hg^2+^ ions or liquid Hg can be released due to the presence of dissolved oxygen or by microbial action, thus contaminating the environment where the electrodes are used. Due to its toxicity, mercury electrodes are not recommended for field analyses [10]. In recent years, development and evaluation of new electrodes, less contaminating than the Hg ones, have been encouraged and some alternatives have been proposed. Amongst them, solid amalgams or membrane-covered electrodes can be found [11]. Nafion is a convenient membrane used for the electrode´s modification during the last few years. It consists of a net-type cationic exchanger polymer capable of metallic particles sheltering. Its use was first published by Xing et al. [12]. Xing showed that Nafion prevents particle agglomeration giving chemical stability to the electrode´s surface and it is environmentally friendly. Deposition of Ag nanoparticles on the Nafion film results in a controlled augmentation of the electrode surface, enhancing sensitivity and reproducibility in the detection of Cr (VI) [13,14]. Nafion-covered glassy carbon electrodes, modified with Ag–Hg nanoparticles, have been used for CRp detection [15]. However, mechanical instability due to poor adhesion of the Nafion film to the glassy carbon surface is a clear disadvantage of those electrodes. To avoid this disadvantage and considering that Nafion films show better adhesion to copper than to glassy carbon, copper electrodes covered with a Nafion film have been used for simultaneous determination of Cd and Pb [14,16].

This paper discusses the development and evaluation of Nafion-covered Cu electrodes modified by deposition of AgHg and AgBi bimetallic particles. Bismuth, as a modifying particle, is studied as an alternative to the use of Hg in order to produce a valuable electrode free from mercury. We compare the two electrodes, the one with Hg and the one with Bi, and report on the convenience, from the analytical and toxicity points of view, of using these electrodes. Their use, along with the Brdička reaction, is also addressed [15,17,18]. The modified electrodes were evaluated by cyclic voltammetry (CV), electrochemical impedance spectroscopy (EIS), and scanning electron microscopy (SEM) [19]. After characterization, the electrodes were used in the laboratory as electrochemical sensors for cysteine and glutathione determination. These two peptides were chosen as model analytes of the peptides released by plants when they are under heavy metal stress [20]. Finally, to check the performance of both the AgHgNf/Cu and the AgBiNf/Cu modified electrodes in real samples, they were applied to the determination of CRp generated by *Nicotiana tabacum* sample cells exposed to cytotoxic levels of Cd [4].

## 2. Experimental

### 2.1. Reagents

All reagents were analytic grade. l-cysteine and Nafion 5% (*w*/*w*) were purchased from Sigma-Aldrich, (Darmstadt, Germany). Nitric acid, hydrochloric acid, and potassium nitrate were purchased from Fisher Scientific (Hampton, NH, USA). Ammonia solution, *N*,*N* dimethylformamide (DMF), 1000 mg L^−1^ Hg(II) standard aqueous solution, cobalt nitrate hexahydrate, silver nitrate, bismuth nitrate, and mercury nitrate were purchased from Merck (Darmstadt, Germany). l-glutathione reduced was purchased from AppliChem (Darmstadt, Germany). All solutions were prepared with distilled/deionized water, 18 MΩ cm^−1^.

### 2.2. Instrumentation

Voltammograms and electrochemical impedance spectra were obtained using a Bio-Logic SP-200 potentiostat (Seyssinet-Pariset, France) interfaced to a computer system with EC-Lab software V11.26 (Seyssinet-Pariset, France), and a standard cell (25 mL volume) with three electrodes. Working electrodes were pure copper (Cu), copper modified with a Nafion film (Cu/Nf), copper modified with silver and mercury bimetallic nanoparticles on a Nafion film (AgHgNf/Cu), and copper modified with silver and bismuth bimetallic nanoparticles on a Nafion film (AgBiNf/Cu). The reference electrode was the Ag/AgCl (3 mol L^−1^ KCl). A platinum wire was used as an auxiliary electrode. SEM images were performed using a PHENOM PROX electronic microscope (Hurk, Netherlands), coupled to an X-ray microanalyzer (Hurk, Netherlands).

### 2.3. Electrode Development

#### 2.3.1. Electrode Construction

The copper electrode consists of a Cu wire, 8 cm long and 3 mm diameter inserted into a 6 cm long, 6 mm diameter glass tube. Filling the glass tube with Rally epoxy resin after insertion of the wire allowed us to keep the wire fixed inside the tube and to avoid possible filtering of any liquid in which the electrode was immersed. After addition of the resin, the electrode dried at room temperature for 12 h. The lower end of the electrode was mechanically polished with sandpaper No. 2000 until the tip of the Cu wire was completely exposed. Polishing the exposed copper surface continued in two steps, firstly using a 1200 grain size silicon carbide disk and secondly using a 0.05 µm grain size alumina powder until a mirror-like Cu surface was obtained. After this, the electrode was sonicated for 5 min and washed with water to eliminate any alumina excess. Finally, the electrode was oven dried at 40 °C for 10 min. [16]. The length of copper wire protruding from the other end of the electrode was used to connect the electrode to the electrochemical cell.

#### 2.3.2. Bimetallic Deposits Formation

A 5 µL volume of a 1% Nafion solution and 3 µL of pure DMF were sequentially and slowly cast on the cleaned electrode surface while it was mechanically rotated at 80 rpm during 30 min [21]. When the Nafion film was formed, the electrode was immersed in a 0.85 mg·L^−1^ Ag^+^ and a 0.15 mg·L^−1^ Hg^2+^ or Bi^3+^ standard solution during 180 min [15]. Then, the electrode was removed from the solution and sprayed with clean-deionized water to wash out non-adsorbed solutions. Finally, to reduce ions trapped inside the Nafion film, the electrode was submitted to controlled potential coulometry (CPC) at −1.2 V for 300 s in a solution containing 1 mol L^−1^ KNO_3_ and 0.1 mol L^−1^ HNO_3_ [15].

### 2.4. Nicotiana tabacum Plants and Cells Culturing

Tobacco plants were grown in a standard Murashige and Skoog (MS) solid medium supplemented with 1-naphthaleneacetic acid (ANA) and 6-Benzylaminopurine (BAP) solution in a 10:1 ratio) [22]. Plants were incubated at 25 °C and 80% humidity. When seeds were used, the MS solid medium contained only 2,4-dichlorophenoxyacetic acid (2,4-d) [23]. When tobacco cells sprouted, they were transferred to a 500 mL glass flask continuously shaken at 85 rpm to obtain a uniform suspension and sub-cultured once a month to enhance cellular growth. Once constant cellular growth was achieved, the cells were subcultured in three MS media with solutions of 10, 50, and 100 µg L^−1^ CdSO_4_ to initiate cysteine-rich peptide generation. Close follow-up of the cells during these experiments showed that after three days of exposure to CdSO_4_, there was no measurable production of GSH, showing that cells died after this exposure time. However, as the main goal of this assay was to obtain the maximal amount of CRp produced by cells before their death, they were left two more days exposed to the cadmium solution. After 5 days of cellular exposure to CdSO_4_, the cells were washed with a 0.14 mol L^−1^ NaCl, 3 mmol L^−1^ KCl, and 4 mmol L^−1^ Na_2_PO_4_, pH 7.4 solution, manually homogenized and centrifuged at 6000 rpm for 30 min in order to collect all the CRp produced. Then, a 0.5 mL of the suspension formed was taken and diluted to 10 mL with the Brdička supporting electrolyte containing 0.1 mol L^−1^ ammonium chloride and 1 mmol L^−1^ cobalt nitrate hexahydrate solution at pH 9.5. The suspension was then cooled at 6 ± 2 °C, to get it ready for electrochemical measurements of its cysteine and glutathione content [4].

### 2.5. Analytical Procedure

For cysteine-rich peptide, CRp, detection, differential pulse voltammetry (DPV) measurements were carried out in a Brdička supporting electrolyte solution (0.1 mol L^−1^ ammonium chloride buffer solution, pH 9.5, and 1 mmol L^−1^ cobalt nitrate hexahydrate cooled to 6 ± 2 °C), under the following settings: 0.5 V as initial potential and −1.2 V as final potential; a pulse height of 2.5 mV, a step height of −5 mV, and a scan rate of 25 mV s^−1^. Calibration curves were obtained using standard solutions of l-cysteine and glutathione at different concentrations [23,24]. Appropriate standard test solutions of glutathione were added to the cell and DPV measurements were carried out. Quantitation of CRp in real samples of *Nicotiana tabacum* cells exposed to cytotoxic levels of cadmium was done by measuring the Brdička reaction current against the standard calibration curves.

## 3. Results and Discussion

### 3.1. Electrode Characterization

#### 3.1.1. Scanning Electron Microscopy (SEM)

**Figure 2:**
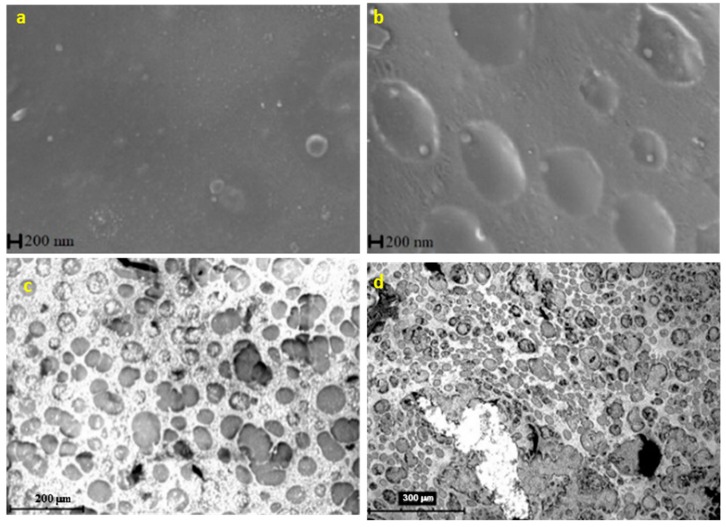
SEM images of (**a**) micrographic view of the bare Cu electrode, (**b**) micrographic view of a Nafion-covered Cu electrode, (**c**) micrographic view of the AgHgNf/Cu electrode, and (**d**) micrographic view of the AgBiNf/Cu electrode.

To study the morphology and structure of the bimetallic nanoparticles, SEM images were taken to the bare Cu electrode, to the Nafion-covered (Nf/Cu) electrode, and to the bimetallic modified (AgHgNf/Cu and AgBiNf/Cu) electrodes. Figure 2a shows a micrographic view of the naked Cu electrode. A very flat surface is observed as a result of the polishing process. Figure 2b represents the electrode surface already covered with Nf. Oval spots evenly scattered all around the Cu surface, whose appearance is not as flat as that of the bare Cu electrode, are due to the presence of the Nafion polymer film covering the electrode surface. Micrographs of the AgHgNf/Cu and AgBiNf/Cu surface are shown in Figure 2c,d, respectively. Bimetallic particles (white spots) with 150 nm approximate diameter are observed attached to the Nafion membrane. Since this is a backscattered electron image, the metallic particles are shinier than the Nafion film. The spreading of the shinier surface shows that the metallic nanoparticles are well distributed on the whole electrode surface [15].

#### 3.1.2. Electrode’s Electrochemical Characterization

**Figure 3:**
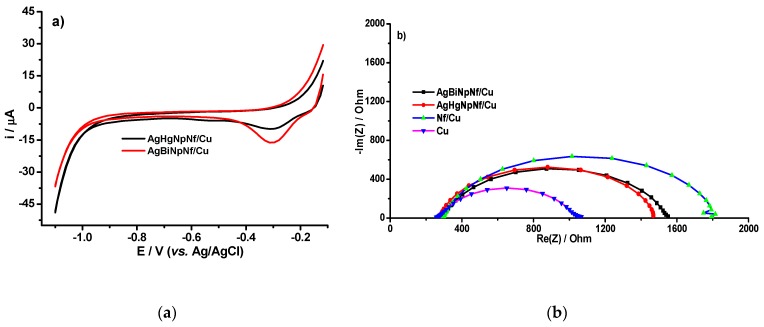
(**a**) Cyclic voltammetry (CV) responses of the AgHgNf/Cu and AgBiNf/Cu electrodes in a 0.1 mol L^−1^ ammonium chloride buffer solution, pH 9.5. Scan rate 0.01 V s^−1^. (**b**) Nyquist impedance of the Cu electrode (purple curve), Nf/Cu electrode (blue curve), AgHgNf/Cu electrode (red curve), and AgBiNf/Cu electrode (black curve) in a 0.1 mol L^−1^ ammonium chloride buffer solution, pH 9.5, at −1.1 V. The frequency range is from 0.1 Hz. to 100 kHz.

Cyclic Voltammetry (CV) curves, for the AgHgNf/Cu and the AgBiNf/Cu, obtained in a 0.1 mol L^−1^ ammonium chloride pH 9.5, are shown in Figure 3a. Both electrodes produce identical curves. It is also evident that, in the voltage region between −0.5 V and −1.1 V, there are no current signals despite the increase in the electrode capacitive current. Since this is the potential region useful to study the Brdička electrochemical phenomena, lack of signals in this region indicates that the electrodes by themselves do not produce any background signals and, in this sense, they are considered clean [16,20]. Figure 3b shows the impedance spectra (EIS) of the bare Cu electrode, the Nf-covered electrode, and the two (AgBiNf/Cu and AgHgNf/Cu) modified electrodes, in an ammonium buffer in the presence of dissolved oxygen. The region of voltage from −0.5 and −1.1 V cover the range of potential used in the reduction of hydrogen [25]. In the plot of impedance spectra, four different semicircles evidence that the process is not influenced by diffusional phenomena. If any diffusion would have occurred, circles in Figure 3b would be distorted. Thus, the figures are definitively due to hydrogen generation [26].

In the EIS, the changes of the electrode superficial resistance can be used to characterize the electron transfer process of the modified electrode. The diameter of the semicircle represents the electron resistance of the electrode, which can be directly measured in the Nyquist impedance curve. The Cu electrode (purple curve), due to his smaller electron resistance, which implies higher electron-transfer capability, has the shortest semicircle´s diameter compared to the other electrodes. On the other hand, the Nf/Cu electrode (blue curve) exhibits the largest semicircle, i.e., the largest electron resistance. This can be attributed to the presence of the Nafion film which increases electron resistance because the anionic species are not capable of passing through the Nafion layer, thus causing charge separation [21]. For the AgHgNf/Cu electrode (red curve) and AgBiNf/Cu electrode (black curve), even though they have the same Nafion cover as the Nf/Cu electrode, the presence of the bimetallic particles increases their surface area and activates the electrodes toward the electrochemical processes causing their electron resistance to become smaller than that of the Nf/Cu electrode. However, the effect of the bimetallic particles is not strong enough to reduce the electron resistance of the bimetallic modified electrodes to a value close to or below that of the bare Cu electrode [14]. In addition, this can be corroborated with the Bode curves, see Supplementary Materials (Figure S1).

### 3.2. Differential Pulse Voltammetry of L-cysteine and Co(II) Ions using the AgHgNf/Cu and AgBiNf/Cu Modified Electrodes

**Figure 4:**
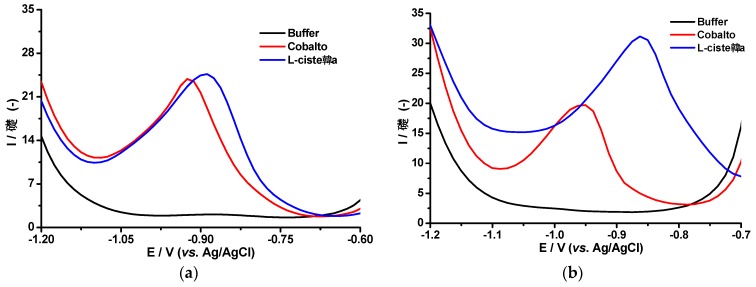
Differential pulse voltammetry (DPV) responses of the AgBiNpNf/Cu (**a**) and AgHgNpNf/Cu (**b**) modified electrode, in 0.1 mol L^−1^ ammonium chloride buffer solution, pH 9.5, (black curve), of the Brdička supporting electrolyte in the absence (red curve) and in the presence of l-cysteine (blue curve), 25 mV s^−1^ scan rate.

Differential pulse voltammetry responses of the Brdička supporting electrolyte in the absence and the presence of L-cysteine using the AgBiNf/Cu modified electrode is displayed in Figure 4a. A reduction peak at −0.93 V (red curve), attributable to Co, was also observed. After addition of the l-cysteine peptide, the Co reduction peak was shifted about 0.2 V towards less negative potentials (blue curve). This behavior could be associated with the formation of the Co–Cys complex and reduction of Co from the complex, which requires less energy than its reduction in the absence of the peptide [20]. Similar behavior was observed using the AgHgNf/Cu electrode (Figure 4b).

**Figure 5:**
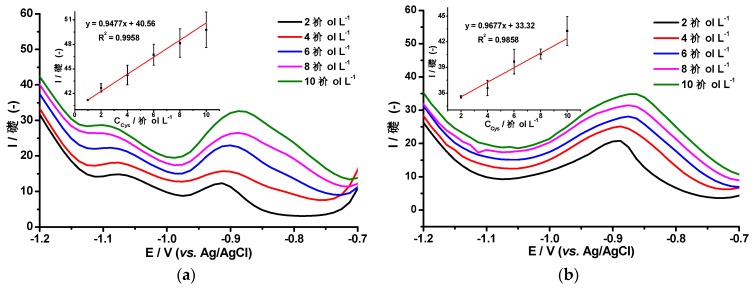
VPD responses of (**a**) AgHgNf/Cu electrode and (**b**) AgBiNf/Cu electrode in the Brdička supporting electrolyte after addition of 2 to 10 µmol L^−1^ of l-cysteine. Inset: Calibration curves of l-cysteine at the modified electrodes. 25 mV s^−1^ scan rate.

Brdička signals were used to detect low molecular weight cysteine-rich peptides based on the linear dependence between the peptide concentration and the hydrogen evolution signals as shown in Figure 5 [27]. This figure shows the DPV responses of AgHgNf/Cu (Figure 5a) and AgBiNf/Cu (Figure 5b) electrodes according to the Brdička reaction in the presence of different concentrations of Cys. It is clearly seen that the hydrogen evolution signal at −1.25 V follows a linear dependence on the cysteine concentration in the range from 1 to 10 µmol L^−1^ of the peptide (Insets Figure 5). The Co peak current shift towards less negative potentials and the signal from the catalytic hydrogen evolution, HE, are characteristics of a Brdička behavior. The variability of measurements of a sample of the electrolytic solution (2.4% RDS) and the three-sigma criterion method was used for determination of the precision and the detection and quantification Limits (DL and QL, respectively) [28]. For the AgHgNf/Cu modified electrode (Inset, Figure 5a) DL and QL values were 0.088 µmol L^−1^ and 0.29 µmol L^−1^, respectively. Whereas for the AgBiNf/Cu modified electrode, a DL of 0.41 µmol L^−1^ and a QL of 1.35 µmol L^−1^ were obtained.

Figure 5a reveals another catalytic phenomenon in the AgHgNf/Cu response. A wave, not present in Figure 5b, appears at −1.1 V. This wave has been previously reported as “Peak P” in works performed using hanging drop mercury electrodes (HDME) based on the Brdička system. It has been shown to be thiol-dependent [8]. Presence of the “Peak P” in the curves obtained using the AgHgNf/Cu electrode shows that the new bimetallic modified electrode performs in a similar way as HDME electrodes do, with similar catalytic response and similar linear dependence (R^2^ = 0.9958) but with considerably less probability of contamination due to its substantially significant reduction in Hg content. This also indicates that the Hg nanoparticles supported in the Nafion film are electrochemically active. Clearly, the new AgHgNf/Cu electrode, even though it is not completely free from mercury, represents a considerable advance in regard to the use of a lesser contaminating sensor as compared to the HDME or to any other massive bare Hg electrode. The absence of “Peak P” on the curves obtained using the AgBiNf/Cu electrode could be ascribed to the absence of mercury on its surface. The important fact here is that these results show that Bi could conveniently replace Hg in the electrode surface since the analytic performance of the AgBiNf/Cu electrode, is close to that of the AgHgNf/Cu. The difference between the two slopes of the curves is approximately 19%, indicating a slightly higher sensitivity of the electrode containing mercury (See insets) (Figure 5). With the exception of this minor loss in sensitivity, the AgBiNf/Cu electrode could be as useful as the electrodes containing Hg and represents a valid option as a sensor which is totally free from mercury.

### 3.3. Differential Pulse Voltammetry of Glutathione and Co(II) Ions Using the AgHgNf/Cu and the AgBiNf/Cu Modified Electrodes

**Figure 6:**
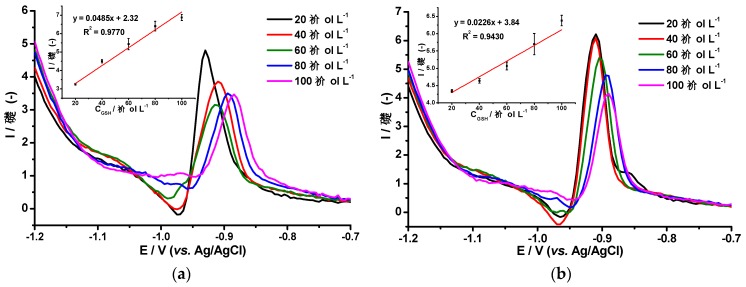
VPD responses of (**a**) AgHgNf/Cu electrode and (**b**) AgBiNf/Cu electrode in a Brdička supporting electrolyte after addition of 20 to 100 µmol L^−1^ of Glutathione (GSH). Inset: Calibration curves of GSH using the AgHgNf/Cu and the AgBiNf/Cu electrodes. 25 mV s^−1^ scan rate.

It has been reported that the GSH tripeptide is capable of heavy metal encapsulation and can be used as a model analyte of cysteine-rich peptides [5]. Glutathione is an interesting molecule due to the various functions it performs in biological systems. Its role as an antioxidant, along with its electrochemical behavior, have been widely studied and reported in the past few years [29]. Regarding electrochemistry aspects, some of these publications deal with electrode modifications made with the purpose of improving the analytical detection of GHS. We could take, as an example, a work reported on the use of multiwall carbon nanotubes coupled to electrochemical pulse techniques to detect glutathione. The paper mentions detection limits below 1 µmol L^−1^ and a linear interval between 0.3 µmol L^−1^ and 18.3 µmol L^−1^ [30]. Another paper reports similar sensitivity with CV and SWV techniques [29]. The sensitivity obtained by these methods can be explained on the base that GSH has a dual linear response, one response at low concentration (~1 µmol L^−1^), and another one at higher concentrations. This creates the already-mentioned electrode partial saturation, which is common when working with biological systems [4,15]. Our work deals with the study of the electrochemical responses of the newly developed AgHgNf/Cu and the AgBiNf/Cu electrodes, coupled to a differential pulse voltammetry system as a way to determine the amount of GSH generated by plants under heavy metal contamination; Figure 6 shows such responses. As observed, a current peak appears at −0.93 V, attributable to the reduction of the Co–GSH complex by the hydrogen evolved as a result of catalysis according to the Brdička reaction. Other researchers have reported the appearance of this peak at −0.93 V, which increases as a function of Cysteine or GSH addition [15,20,27]. Contrary to those findings, signals in Figure 6 decrease with increasing GSH concentration. These results can be explained considering the size of the Glutathione molecule. The GSH, being a tripeptide, is a molecule much larger than cysteine and when GSH reacts with Cobalt to produce the Co–GSH complex, the complex could form an electrostatic monolayer on the electrode´s surface. Formation of the Co–GSH monolayer could produce partial saturation of the sensor´s surface with the consequent repulsion of new GSH molecules in that particular area of the electrode. Under this condition, lesser amounts of GSH reach the electrode leading to less Co–GSH complex formation with the consequent size reduction of the Co–GSH peak. Even though the Co–cysteine complex could also be formed, the Co–cysteine complex does not seem to affect the electrode´s area probably due to the smaller size of this complex molecule. Excluding the slighter less sensitivity of the AgBiNf/Cu electrode, both electrodes behave roughly in a similar fashion as regards to their responses to the reduction of the Co–GSH as shown in Figure 6. It is evident from Figure 6 that the relationship between I and E and their dependence on the amount of GSH added is kept at least up to the addition of 100 µmol L^−1^. Data corresponding to Figure 6 were used to calculate detection limits for both electrodes. Using the AgHgNf/Cu electrode, a detection limit of 0.139 µmol L^−1^ and a quantitation limit of 0.4622 µmol L^−1^ were obtained. The corresponding values for the AgBiNpNf/Cu electrode are 0.244 µmol L^−1^ and 0.813 µmol L^−1^ [28].

### 3.4. Determination of CRp Generated by Nicotiana tabacum Cells Exposed to Cytotoxic Levels of Cd

**Figure 7:**
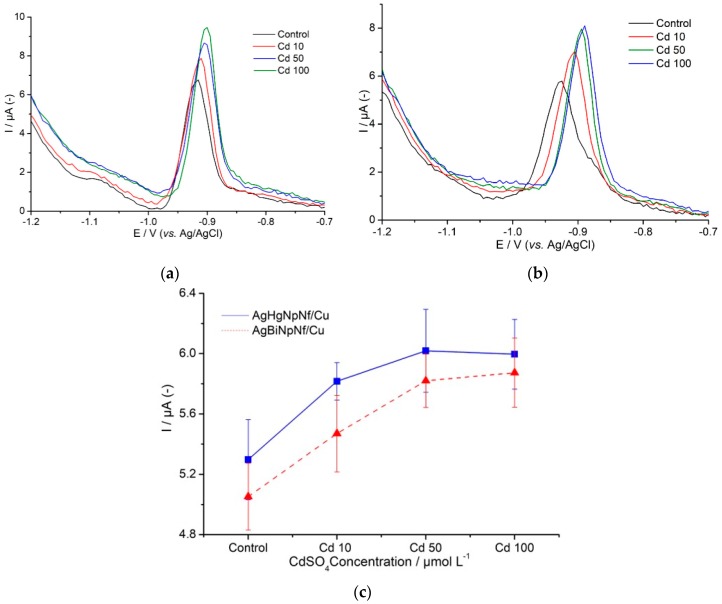
Differential pulse voltammetry signals obtained during the experiments: (**a**) AgHgNpNf/Cu electrode and (**b**) AgBiNpNf/Cu electrode. (**c**) Dependence of the HE current generated by nicotiana tabacum cells on increasing concentration of Cadmium sulfate in a Brdicka medium using the AgBiNf/Cu, and the AgHgNf/Cu electrodes in a Brdička medium, after 5 days culturing.

Signals of catalytic hydrogen evolution (HE) based on the Bridcka reaction were used as analytical signals to follow-up the generation of CRp in tobacco cells, *Nicotiana tabacum*, suspensions cultured in vitro in the absence, taken as blank signals, and in the presence of 10, 50, and 100 µmol L^−1^ of CdSO_4_ solutions. Each cell suspension was exposed for one, two, three, and five days to each one of the CdSO_4_ solutions. Figure 7a,b show the differential pulse voltammetry signals obtained during these experiments. The figures indicate that culture time of cells in the absence of CdSO_4_ is not an influential factor on the intensity of the HE signals at around 1.2 V. The main factors influencing the intensity of the HE signals are the presence of Cd, concentration, and the exposure time. The intensity of the signals increases as long as Cd concentration in the medium increases up to 50 µmol L^−1^. Doubling the amount of CdSO_4_ from this value does not show a significant increment in the intensity of the signals. This behavior clearly indicates that presence of Cd promotes CRp generation and the amount of CRp generated is depending on the amount of Cd in accordance with the fact that this is a plant´s reaction aiming at its defense against contamination by the heavy metal [1]. Our study also demonstrates that Cd exposure time is another factor to take into consideration (Figure 7c). Exposure for just one day, even to the lowest CdSO_4_ solution, clearly promoted CRp generation. Generation of enough cysteine-rich peptides is not an instantaneous process, and production of sufficient amounts to protect the plant and amenable to be detected must take some time. For an effective plant´s protection response, the phenomenon must initiate at the first moment when the *Nicotiana tabacum* cells enter in contact with the contaminant and must continue until some cell mechanism stops it. In our essays, at low Cd concentrations, in a two-day exposure, the amount of CRp produced seems to be enough to control the effect of that particular amount of contaminant and allow cells to continue their development. Exposure lasting three days at high Cd concentration (50 µmol L^−1^) showed CRp generation to reach a plateau after which it stops and is followed by a brisk jump which is typical of an apoptosis stage. When the contaminant solution contains 100 µmol L^−1^ CdSO_4_, CRp generation is complete in just two days. The plateau is reached as indicated by HE signals becoming constant but there is no further indication of changing with time. We assume that at this moment, the plant is no longer able to produce more CRp to defend itself from that relatively vast amount of contaminant, leading to irreversible cellular death. This behavior is represented in Figure 7. Performance reproducibility of the electrodes was ascertained by preparing four modified AgHgNf/Cu and four modified AgBiNf/Cu electrodes and measuring the coefficients of variation, CV, of the slopes of four calibration curves, each one obtained with each electrode. For the corresponding calibration curves, a coefficient of variation of 7% was obtained. The electrodes were then stored for eight days at room temperature. One of them was randomly taken to study its repeatability by comparing the slopes of four calibration curves obtained with the same electrode. These measurements permit calculation of a VC value of 3%.

## 4. Conclusions

This work describes the development and evaluation of two new types of electrodes consisting of a copper wire, inserted in a glass tubing, covered by a Nafion film in which particles of bimetallic combinations of AgHg (the AgHgNf/Cu electrode) and AgBi (the AgBiNf/Cu electrode) were deposited. The study shows the viability and convenience of using these electrodes, via the Brdička reaction, as analytical tools for diagnosis of plants heavy metal contamination. The diagnosis is accomplished by quantifying the amounts of cysteine-rich peptides produced by the plant as a response to the contamination stress. *Nicotiana tabacum* live cells were used as model plants and Cd as a representative of a contaminant metal. The AgHgNf/Cu electrode was found to produce analytical responses similar to those obtained by the HDME or any other massive Hg electrode with the advantage of being less contaminant due to its substantial reduction in mercury content. The AgBiNf/Cu electrode performed in a similar way as the AgHgNf/Cu one, somehow less sensitive, but with the added advantage of being totally free from mercury. Both electrodes coupled to a DPV system could be used to perform in situ diagnostics of eco-systems and detection of heavy metal vegetation contamination in a simple and rapid way with no risk of mercury contamination to the environment.

## Supplementary Materials

The following are available online at https://www.mdpi.com/1420-3049/24/12/2200/s1, Figure S1: Bode curves impedance of the Cu electrode (blue), Nf/Cu electrode (red), AgHgNf/Cu electrode (purple) and AgBiNf/Cu electrode (red) in a 0.1 mol L^−1^ ammonium chloride buffer solution, pH 9.5, at −1.1 V. The frequency range is from 0.1 Hz. to 100 kHz.
