# Supplementary material for: Development and Evaluation of Copper Electrodes, Modified with Bimetallic Nanoparticles, to be Used as Sensors of Cysteine-Rich Peptides Synthesized by Tobacco Cells Exposed to Cytotoxic Levels of Cadmium
Source: Molecules. 2019 Jun 12;24(12):2200. doi: 10.3390/molecules24122200 (PMC6631066; doi:10.3390/molecules24122200)
Supplement: Supplementary file 1 [file molecules-24-02200-s001.pdf]

Supplementary Materials

BODE IMPEDANCE GRAPHS

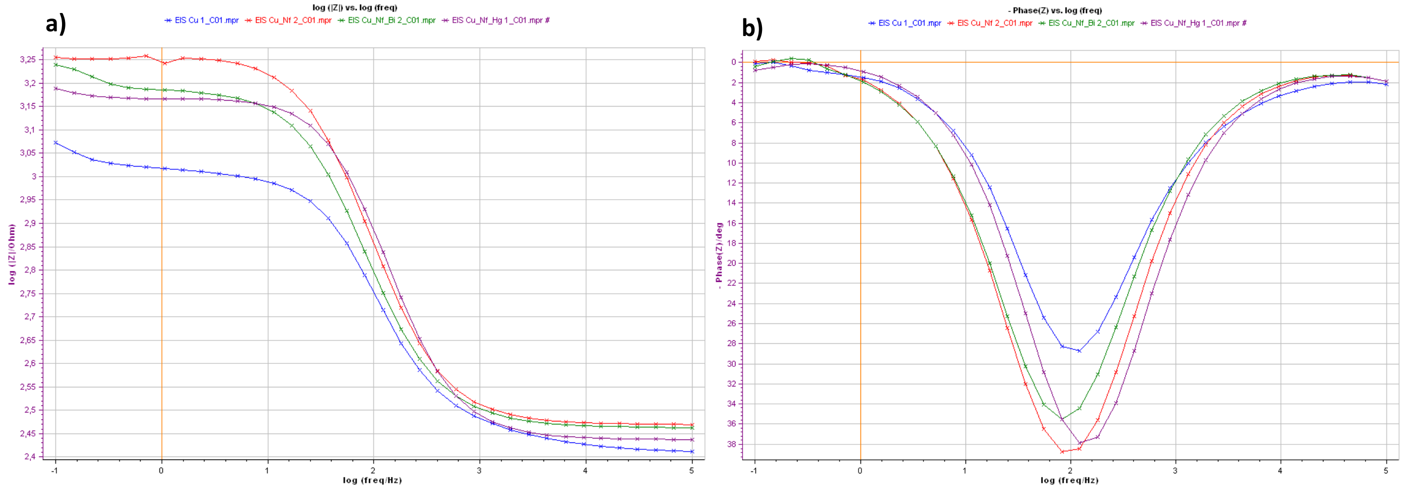

Figure S1. a) Bode curves impedance (Log |z| *vs.* Log *freq*) ; **b)** Phase angle as a function of Log freq. Cu electrode (blue), Nf/Cu electrode (red), AgHgNf/Cu electrode (purple) and AgBiNf/Cu electrode (red) in a 0.1 mol L^{-1} ammonium chloride buffer solution, pH 9.5, at -1.1 V. The frequency range is from 0.1 Hz. to 100 kHz.

In Figure S1a, we can see the impedance of the Nafion modified Cu electrodes and the naked Cu electrode *vs.* frequency. At -1,1 V and low frequencies where the faradic reaction of hydrogen evolution is taking place. The effect of the added nanoparticles is evidently diminishing the impedance of the whole electrochemical process comparing with the Cu Nafion electrode. In Figure S1b, we confirm the presence of one RC element which lead us to propose an equivalent circuit for the electrochemical process:

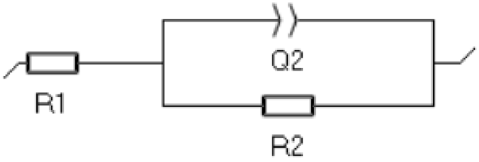

Figure S2. Equivalent Circuit: R1+Q2/R2; R1: ELECTROLYTE RESISTENCE; Q2: DOUBLE LAYER CAPACITANCE; R2: CHARGE TRANSFER RESISTENCE
